# The Physical Activity Wearables in the Police Force (PAW-Force) study: acceptability and impact

**DOI:** 10.1186/s12889-020-09776-1

**Published:** 2020-11-03

**Authors:** Sarah Ann Buckingham, Karyn Morrissey, Andrew James Williams, Lisa Price, John Harrison

**Affiliations:** 1 European Centre for Environment and Human Health, Knowledge Spa, Royal Cornwall Hospitals NHS Trust, University of Exeter Medical School, 2nd Floor Knowledge Spa, Royal Cornwall Hospital, Truro, TR1 3HD UK; 2 Population and Behavioural Science, School of Medicine, University of St Andrews, St Andrews, UK; 3 Sport and Health Sciences, College of Life and Environmental Sciences, University of Exeter, Exeter, UK; 4 Occupational Health Support Unit, Devon and Cornwall Police, Middlemoor, Exeter, UK

**Keywords:** Mobile health, Physical activity, Sedentary behaviour, Police force, Behaviour change

## Abstract

**Background:**

Policing is a highly stressful and increasingly sedentary occupation. The study aim was to assess the acceptability and impact of a mobile health (mHealth) technology intervention (Fitbit® activity monitor and ‘Bupa Boost’ smartphone app) to promote physical activity (PA) and reduce sedentary time in the police force.

**Methods:**

Single-group, pre-post, mixed methods pilot study. Police officers and staff (*n* = 180) were recruited from two police forces in South West England. Participants used the technology for 12 weeks (an ‘individual’ then ‘social’ phase) followed by 5 months of optional use. Data sources included Fitbit®-recorded objective step count, questionnaire surveys and semi-structured interviews (*n* = 32). Outcome assessment points were baseline (week 0), mid-intervention (week 6), post-intervention (week 12) and follow-up (month 8). Paired t-tests were used to investigate changes in quantitative outcomes. Qualitative analysis involved framework and thematic analysis.

**Results:**

Changes in mean daily step count were non-significant (*p* > 0.05), but self-reported PA increased in the short term (e.g. + 465.4 MET-minutes/week total PA baseline to week 12, *p* = 0.011) and longer term (e.g. + 420.5 MET-minutes/week moderate-to-vigorous PA baseline to month 8, *p* = 0.024). The greatest impact on behaviour was perceived by less active officers and staff. There were no significant changes in sedentary time; the qualitative findings highlighted the importance of context and external influences on behaviour. There were no statistically significant changes (all *p*-values > 0.05) in any secondary outcomes (physical and mental health-related quality of life, perceived stress and perceived productivity), with the exception of an improvement in mental health-related quality of life (SF-12 mental component score + 1.75 points, *p* = 0.020) from baseline to month 8. Engagement with and perceived acceptability of the intervention was high overall, but a small number of participants reported negative physical (skin irritation) and psychological (feelings of guilt and anxiety) consequences of technology use. Individual app features (such as goal-setting and self-monitoring) were generally preferred to social components (social comparison, competitions and support).

**Conclusions:**

mHealth technology is an acceptable and potentially impactful intervention for increasing PA in the police force. The intervention was less useful for reducing sedentary time and the impact on secondary outcomes is unclear.

**Trial registration:**

NCT03169179 (registered 30th May 2017).

**Supplementary Information:**

**Supplementary information** accompanies this paper at 10.1186/s12889-020-09776-1.

## Background

Policing is an increasingly sedentary occupation [1], due in part to the changing nature of policing, with increasing rates of white collar and cybercrime relative to street crime [2]. Police officers have been shown to be more active on their off-duty days than when they are at work [1]. Policing is also a highly stressful occupation; police officers (and also staff) are exposed to a range of acute and chronic stressors as part of their role, in addition to organisational pressures [3]. Additional lifestyle issues associated with the policing occupation include shift work, poor sleep and unhealthy diets [4].

Studies in various countries have indicated that the police force has a high prevalence of cardiovascular risk factors and health conditions, including high body mass index (BMI) and obesity [5, 6], metabolic syndrome [7, 8] and cardiovascular disease [9–11]. Evidence suggests that the prevalence of these conditions is higher than in the general population [10–12], with lifestyle factors such as physical inactivity, stress and shift work thought to be contributing factors [9, 11].

A physically active lifestyle may potentially mitigate the health risks associated with the policing occupation [6, 8]. In addition to enhancing the wellbeing of individuals, there may be potential organisational benefits such as improved productivity and reduced absenteeism [13, 14]. Furthermore, police officers need to meet professional standards of fitness in order to pass the annual fitness test and deal with situations that necessitate fitness, endurance or the use of physical force.

The need for novel interventions to promote physical activity (PA) and reduce sedentary behaviour (SB) in the police force has been emphasised [1, 8, 15]. Mobile health (mHealth) technology, including wearable PA monitors or trackers and smartphone applications or apps, is increasingly popular and potentially of high value in health promotion due to its widespread appeal, accessibility, scalability and cost-effectiveness [16]. Technology typically contains behaviour change techniques (BCTs), which may incorporate evidence-based behaviour change principles [17].

Despite its considerable potential, there are many gaps in our understanding of how mHealth technology may be utilised to change behaviour and its potential impact on health outcomes. Systematic reviews have found mHealth studies to be characterised by short-term interventions and follow-up [18–21], with follow-up typically 6 months or less [22–25] and often only a few weeks [26–30] in duration. Aspects such as feasibility, acceptability and engagement are of vital importance, but remain understudied in mHealth research [31]. The longer-term impact and acceptability for promoting PA, the impact on SB, and the impact on wider outcomes such as health, wellbeing and productivity, are unclear [21]. Furthermore, reviews have found that many mHealth interventions are neither theory-based nor evidence-informed [18, 19, 32, 33]. As a result, it is unclear which components (e.g. app features) may be most impactful for changing behaviour. For example, studies that have explored the relative impact of ‘individual’ and ‘social’ app features for increasing PA have reached opposing findings [34, 35].

To address the gaps in evidence, the aim of the Physical Activity Wearables in the Police Force (PAW-Force) study was to explore the feasibility, acceptability and impact of an mHealth intervention (consisting of a Fitbit® activity monitor and ‘Bupa Boost’ smartphone app) to promote PA and reduce SB in the police force, in the short and longer term. The objectives of this mixed methods study were: to assess the impact on PA, sedentary time and secondary outcomes (health and wellbeing, perceived stress, perceived productivity and sickness absence); to explore acceptability and engagement with the intervention; and to identify preferred and impactful intervention components.

## Methods

### Overview of study design and context

The study used a single group, pre-post and follow-up, mixed methods pilot design. Participants received a 12-week mHealth intervention with 8 months to follow-up (June 2017 to February 2018). The study was part of a three-year wellness programme (January 2017 to January 2020) within the Devon and Cornwall Police and Dorset Police forces (‘ActivAte 2020’). The programme included a number of initiatives to target PA, diet and nutrition, and sleep quality. These included written information and screensaver campaigns on health and wellbeing, deployment of health and wellbeing champions, ‘healthy weight’ diagnostic sessions and taster sessions with sports clubs. These initiatives complemented the mHealth intervention (the Fitbit® and Bupa Boost app) which was the main initiative that specifically aimed to increase PA. The mHealth intervention was not designed by the research team, but was pre-determined and provided by the police forces involved.

Ethical approval was granted by the University of Exeter Medical School Research Ethics Committee prior to study commencement (Ref. 17/02/116). The study protocol was registered with ClincalTrials.gov (Ref. NCT03169179).

### Participants

Police officers and staff were recruited from two sites in South West England - Plymouth Basic Command Unit (Devon and Cornwall Police) and North Dorset territorial area (Dorset Police). The target sample size (*n* = 180) was based on a power calculation for the primary quantitative outcome variable (mean daily step count), expected attrition and feasibility of resources.

All participants were volunteers, recruited using a combination of initial convenience sampling and later purposive maximal variation sampling to ensure sufficient representation of different occupational groups and work streams (including local investigation, local/neighbourhood policing, response policing, intelligence, communications and administration). The study was advertised using posters in the workplace, police force intranets, staff bulletins and e-mails throughout the organisations. As an inclusive wellness programme, officers in a range of ranks (constable, sergeant, inspector and higher level officers), Police Community Support Officers (PCSOs), special constables, and police staff (mostly office-based) were all invited to participate.

Interested participants were provided with an information sheet and completed online screening and consent forms. Inclusion criteria were officers and staff who expected to be employed within the police force for the duration of the study, and who owned (or had access to) a smartphone or tablet that was compatible with the Bupa Boost app (Apple or Android 4.0.3 or higher), with Bluetooth and internet access. The only exclusion criterion was severe limited mobility, i.e. those who would be physically unable to increase their step count over the duration of the study.

### Intervention and study process

**Fig. 1:**
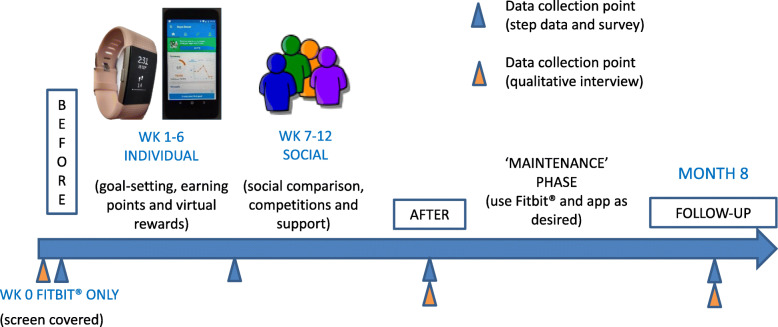
Overview of the study process. Note: All images are the authors’ own

All participants were provided with a Fitbit® Charge 2 activity monitor, which they were able to use free of charge as long as they remained employed by the police forces involved in the study. The Bupa Boost app, which is run by the private health insurer Bupa and specifically designed to promote health and wellbeing in the workplace, was also provided free of charge. The Fitbit® was able to synchronise with the Bupa Boost app. The intervention was coded using the CALO-RE (Coventry, Aberdeen and London – Refined) taxonomy, a standardised classification system of evidence-based BCTs for PA and healthy eating behaviours [36]. Together, the Fitbit® and Bupa Boost app contained 20 of a possible 40 BCTs, which are detailed in Additional File 1.The study process is illustrated in Fig. 1. Officers and staff were instructed to wear the Fitbit® on their wrist for seven consecutive days at baseline (week 0) while maintaining their usual activity levels. The screen was covered by a sticker during this baseline week, and participants were asked not to log in to the Fitbit® app during this time; this helped to ensure a valid pre-intervention measure of the primary quantitative outcome (daily step count). Following completion of the baseline questionnaire, participants entered the intervention phase, where they began to use the Fitbit® (with the screen uncovered) together with the Bupa Boost app. All participants were instructed to increase their daily step count from their baseline level, in addition to increasing their participation in any other physical activities that were of interest to them.

The 12-week intervention was divided into an ‘individual’ and a ‘social’ phase. The purpose of this was to assess the relative impact and acceptability of the different features in a way that was feasible within the apps. During weeks 1 to 6, participants were instructed to only use the ‘individual’ features of the Fitbit® and Bupa Boost app. These included:

- individual (personal) goal-setting;
- self-monitoring;
- feedback on progress via the app;
- earning virtual rewards for achievements (wellness points and badges); and
- access to the ‘Bupa library’ within Bupa Boost, for self-help information on maintaining a healthy lifestyle.

During weeks 7 to 12 (the ‘social’ phase), participants were encouraged to link up with their colleagues within the Bupa Boost app. In addition to the ‘individual’ features, they were able to:

- compare themselves with their colleagues via a social feed;
- compete with their colleagues (in individual challenges and/or working as part of a team in company challenges); and
- give and receive social support through virtual ‘likes’ and messages.

While participants were also able to use the Fitbit® app in the intervention phase, they were they were instructed to use the Bupa Boost app instead of the Fitbit® app for the social features. At the end of the 12 weeks, there was a 5 month ‘maintenance phase’ during which participants continued to use the Fitbit® and Bupa Boost app as and when they desired.

The study was managed remotely, with regular generic written instructions sent via e-mail by the researcher. These included details of how to register, wear and charge the Fitbit® (week 0), how to obtain and upload step count data (week 1), how to use the Bupa Boost app and connect it to the Fitbit® (week 1), which additional features were available during the ‘social’ phase (week 6–7), and where to find further information or support with technical issues. In week 1, participants also received written guidance on setting ‘SMART’ goals (Specific, Measurable, Achievable, Relevant and Time-bound). The intervention was self-directed, delivered with minimal in-person input, as this was the least resource-intensive and most practical approach.

### Outcome measures

The quantitative outcomes were assessed at baseline (week 0), mid-intervention (week 6), post-intervention (week 12) and follow-up (month 8) using objectively recorded data and online questionnaire surveys. The primary outcome measure was mean daily step count, as recorded by the Fitbit®, with the average calculated from the 7 days prior to data collection. Systematic review evidence has demonstrated high validity and inter-device reliability for the outcome of step count for Fitbit® activity monitors when compared with research-grade accelerometers in both laboratory and ‘free-living’ situations [37, 38]. The specific model used in this study has been validated for step count [39]. Step data were downloaded by participants and uploaded to the survey or e-mailed to the researcher. Similar to the approach employed by previous researchers [27, 40] a minimum wear criterion was applied so that mean daily steps were only calculated for participants who had worn the Fitbit® for five or more of the previous 7 days including at least one weekend day, and where more than 500 steps per day were recorded.

The questionnaire surveys included the following secondary outcomes:

- Self-reported PA (total weekly PA and weekly moderate-to-vigorous PA or MVPA) and weekday sedentary time as assessed by the International Physical Activity Questionnaire (IPAQ) short version [41]
- Physical and mental health-related quality of life as assessed by the 12-item Short Form Survey (SF-12) [42]
- Perceived stress as assessed by the 4-item Perceived Stress Scale (PSS-4) [43]
- Self-perceived productivity from the absenteeism and presenteeism questions of the World Health Organisation (WHO) Health and Work Performance Questionnaire (HPQ) [44]

Quantitative and qualitative measures of engagement and acceptability of the intervention were also captured in the surveys. These included self-reported usage of the Fitbit® and Bupa Boost app (wear/usage time, goals set and features used), perceived usability and usefulness (assessed by Likert scale responses on perceived ease of use and perceived usefulness for increasing PA), and reasons for disengagement.

A pre- and post-intervention analysis of sickness absence data (total days lost, duty days lost and reasons for absence) was also specified in the study protocol.

### Interviews

Semi-structured interviews were conducted with a sub-sample of study participants, selected from those who had indicated on the initial consent form that they agreed to be approached for this purpose. Participants were purposively selected for maximal variation according to age, gender and occupation. Purposeful sampling is a widely used method to efficiently select information-rich cases in qualitative research [45]. Where possible, the same participants were interviewed at each time point, but some additional interviewees were selected due to reasons of availability, and a need to ensure representation of those with a range of activity levels and who had shown various levels of engagement with the intervention and behaviour change. Interviews took place at three time points - prior to the intervention (week 0), at post-intervention (week 12), and follow-up (month 8). The purpose of the interviews was to gain in-depth information on participants’ experiences, including perceived impact, engagement with and acceptability of the intervention. The topics covered in the interviews are given in Table 1. In total, 32 interviews were conducted with 16 participants. With the exception of one face-to-face interview, all interviews were conducted via telephone. The interviews were audio-recorded and transcribed verbatim. Field notes were taken during or immediately after each interview and used to guide analysis.

**Table 1:** Overview of topic guide for interviews

Interview	Topics
Pre-intervention (week 0)	Prior experiences and expectations of mHealth / fitness technology
Post-intervention (week 12)	Short-term engagement with the intervention
	Experiences and short-term behaviour change
Follow-up (month 8)	Longer-term engagement and experiences
	Maintenance of PA levels
	Experience of study participation
All interviews	Wider context of PA and SB in the police force (including workplace PA initiatives, use, barriers and suggestions)
	Barriers and facilitators for PA
	Barriers and facilitators for technology use

### Statistical analysis

Descriptive and inferential statistics were calculated for the quantitative data. After checking for normal distributions, paired t-tests were used to assess changes in primary and secondary variables between baseline and week 6, week 12 and month 8. To assess differences in the relative impact of the individual and social phase, the change in PA outcomes (mean daily step count and self-reported PA) and sedentary time between week 0 and week 6 was compared with the change in these outcomes between week 0 and week 12 using paired t-tests. Subgroup analyses were performed for key variables including gender, age group, baseline activity level (< 10,000 steps/day vs. ≥10,000 steps/day) and occupation (officers/staff/PCSOs and special constables). A *p*-value < 0.05 was considered statistically significant and all tests were two-tailed. Quantitative analysis was performed using Stata version 15.0 [46]. With reference to missing data, a combination of complete case analysis and pairwise deletion was used to maximise the amount of data for inclusion.

### Qualitative and mixed methods analysis

Qualitative data were analysed thematically; this process was influenced by theory (deductive) and concepts generated from the data (inductive). The Framework Method [47] was used to systematically organise and analyse interview data. Each code formed a separate column, and each participant formed a separate row of the framework matrix. The cells of the matrix were filled with summaries of the data (including interview content and field notes) and key quotations, allowing comparisons to be made between participants by theme, and within participants over time. Analysis was carried out in NVivo version 11 [48]. While a single researcher performed the coding due to resource limitations, the framework matrices and themes generated were checked by an independent researcher (Dr. C. Guell) to improve rigour.

As a mixed methods study, quantitative and qualitative data were integrated at both the analysis phase and interpretation phase of the study. First, integration took place at an individual participant level within the framework matrix (for example, the inclusion of PA data enabled differences in perceived impact to be explored according to baseline activity levels), and later quantitative and qualitative findings were triangulated at the level of the dataset according to the overall research questions.

## Results

### Participant characteristics

**Fig. 2:**
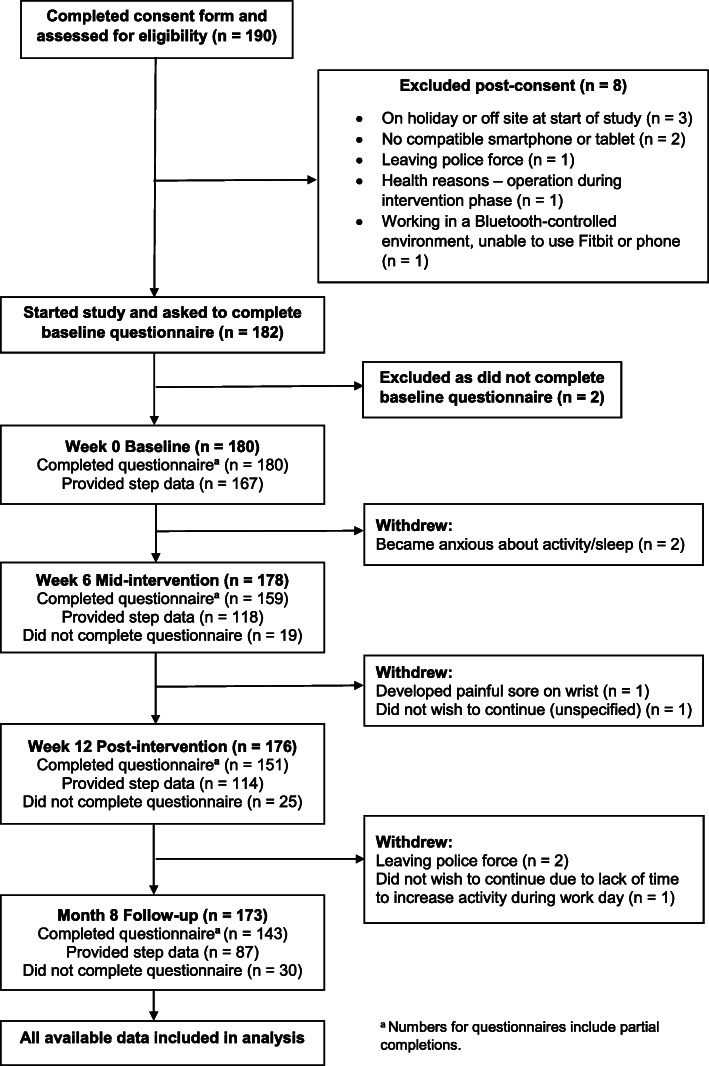
Participant enrolment and retention

The number of participants enrolled in the study and participant flow from initial consent to completion of the final follow-up questionnaire are summarised in Fig. 2. In brief, 190 police officers and staff completed the online screening and consent form; eight of these were ineligible and excluded for reasons shown in Fig. 2. Of the 182 participants beginning the study, 180 provided baseline data. Seven participants officially withdrew through the eight-month study period, and an additional proportion did not complete the questionnaires at each data collection point, i.e. 19/178 (11%) at week 6, 25/176 (14%) at week 12, and 30/173 (17%) at month 8. The overall participant retention rate from beginning the study to 8-month follow-up was 143/182 (79%).

The socio-demographic and occupational characteristics of the 180 participants that provided baseline data are shown in Table 2. Of the total sample, 71% (*n* = 128) were based within the urban Plymouth Basic Command Unit (BCU) and 29% (*n* = 52) were at the more rural North Dorset sites. The age of participants ranged from 19 to 64 years, with a mean age of 39.3 ± 9.6 years. The majority of participants were male, police officers, of White ethnicity, and were shift workers. The sample was diverse in terms of marital status and education. The majority of participants (58%, *n* = 105) reported their role as mainly sedentary compared with only 17% (*n* = 30) who reported being mainly active while on duty, affirming the need for the intervention. Baseline activity levels of officers and staff are shown in Table 3. While the mean daily step count was moderately high at 10,555 steps, there was a large range of activity levels for steps and self-reported PA outcomes. Mean self-reported sedentary time on a typical weekday was 6.41 ± 2.94 h.

**Table 2:** Participant characteristics: socio-demographic and occupational

Study variables	Participated in study (***n*** = 180)
Age in years, mean (SD)	39.3 (9.6)
Male, n (%)	107 (59%)
Ethnicity, n (%)	
White	177 (98%)
Marital status, n (%)	
Single (never married or civil partnered)	40 (22%)
Married or civil partnership	112 (62%)
Divorced, separated or widowed	26 (14%)
Prefer not to say	2 (1%)
Main residence, n (%)	
Urban (city or town)	96 (54%)
Suburban	43 (24%)
Rural (including rural village, hamlet or isolated dwelling)	41 (23%)
Highest level of education, n (%)	
Lower secondary school (GCSE, CSE, O-level, Standard Grade, Intermediates)	38 (21%)
Upper secondary school (AS or A-level, Scottish Highers)	44 (24%)
Professional or technical qualification (below degree)	41 (23%)
University / college degree	48 (27%)
Postgraduate (masters / PhD)	9 (5%)
Police force, n (%)	
Devon & Cornwall Police (Plymouth Basic Command Unit)	128 (71%)
Dorset Police (North Dorset)	52 (29%)
Occupation, n (%)	
Police officer	114 (63%)
Police Community Support Officer (PCSO) or special constable	30 (17%)
Police staff	36 (20%)
Rank, n (%) (officers only, *n* = 114)	
Constable	87 (76%)
Sergeant	23 (20%)
Inspector, chief inspector or superintendent	4 (4%)
Years of police force service, mean (SD)	12.1 (8.0)
Working 30 or more hours per week, n (%)	167 (93%)
Shift work, n (%)	144 (80%)
Type of shift^**a**^ (shift workers only, *n* = 144)	
Morning (early)	95 (66%)
Afternoon (late)	96 (67%)
Night	30 (21%)
Rotating	59 (41%)
How active is your role? n (%)	
Mainly sedentary	105 (58%)
Mainly active	30 (17%)
Equally active and sedentary	45 (25%)

^a^Note: Some participants worked more than one type of shift. *SD* Standard Deviation

**Table 3:** Baseline steps (Fitbit® data), self-reported physical activity (PA) and sedentary time (IPAQ-assessed)

Outcome	n	Mean (SD)	95% CI	Range
Step count (mean steps/day)^a^	167	10,555 (3259)	10,057 to 11,053	3797 to 20,819
Total PA (minutes/week)	180	170.4 (106.2)	154.8 to 186.0	10.0 to 540.0
Total PA (MET-minutes/week)	180	3182.1 (2527.8)	2810.3 to 3553.9	66.0 to 16,398.0
Moderate-to-vigorous PA (MVPA) (MET-minutes/week)	180	1718.6 (1829.5)	1449.5 to 1987.6	0.0 to 12,240.0
Sedentary time (hours on a typical weekday)	180	6.41 (2.94)	5.98 to 6.85	1.00 to 15.00

Note: *n* number of observations; *SD* Standard Deviation; *95% CI* 95% Confidence Interval

^a^ Mean daily steps were calculated for participants who had worn the Fitbit on ≥5 of the previous 7 days including at least one weekend day

Approximately 10% (128/1268) of officers and staff from Plymouth BCU and 71% (52/73) of North Dorset officers and staff participated in the study. Overall, the study participants were representative of the wider police force populations in terms of occupation, gender and ethnicity (see Additional File 2). The characteristics of interviewees are given in Additional File 3.

### Impact on physical activity and sedentary time

As shown in Table 4, there were no significant changes in mean daily step count from baseline to mid-intervention (week 6) or post-intervention (week 12). There was an apparent significant reduction in mean daily step count from baseline to 8-month follow-up (mean decrease 888 steps/day, 95% CI: − 1518 to − 258; *p* = 0.006). However, with a sensitivity analysis including only participants who had reported no events affecting their PA level (such as illness or annual leave) in the previous 7 days, this change became non-significant (mean decrease 765 steps/day, 95% CI: − 1755 to 225; *p* = 0.126) (see Additional File 4).

**Table 4:** Changes in mean daily step count (Fitbit® data) and self-reported physical activity (PA) and sedentary time (IPAQ-assessed) – all participants

Outcome	Mean change from baseline (week 0)								
	Mid-intervention (end of individual phase, week 6)			Post-intervention (end of social phase, week 12)			Follow-up (month 8)		
	Mean change (SD) ***n*** = number of observations	95% CI	***p***-value	Mean change (SD) ***n*** = number of observations	95% CI	***p***-value	Mean change (SD) ***n*** = number of observations	95% CI	***p***-value
Mean daily steps	− 214 (2830) *n* = 118	− 730 to 302	0.413	78 (2599) *n* = 114	− 404 to 561	0.748	−888 (2956) *n* = 87	−1518 to −258	0.006*
Total PA (minutes/week)	27.8 (107.1) *n* = 157	10.9 to 44.7	**0.001**	22.7 (111.2) *n* = 151	4.8 to 40.6	**0.013**	18.6 (113.0) *n* = 143	−0.1 to 37.2	0.052
Total PA (MET-minutes/week)	460.3 (2467.5) *n* = 157	71.3 to 849.3	**0.021**	465.4 (2230.9) *n* = 151	106.7 to 824.1	**0.011**	317.7 (2522.0) *n* = 143	−99.2 to 734.6	0.134
MVPA (MET-minutes/week)	271.9 (1685.1) *n* = 157	6.3 to 537.6	**0.045**	402.9 (1698.0) *n* = 151	129.9 to 676.0	**0.004**	420.5 (2202.6) *n* = 143	56.4 to 784.6	**0.024**
Sedentary time (hours on a typical weekday)	−0.12 (3.36) *n* = 157	−0.65 to 0.41	0.651	−0.03 (3.45) *n* = 151	−0.59 to 0.52	0.906	−0.24 (2.99) *n* = 143	−0.73 to 0.26	0.344

Note: *SD* Standard Deviation; *95% CI* 95% Confidence Interval. Pairwise deletion used

*p*-values where significant (i.e. < 0.05) are highlighted in bold

* The change became non-significant (*p* = 0.126) with a sensitivity analysis controlling for self-reported events affecting PA level (e.g. illness, annual leave) – see Additional File 4

There were significant increases in the self-reported PA outcomes in the short term (see Table 4). From baseline to week 6, total PA increased by a mean of 27.8 min/week (95% CI: 10.9 to 44.7; *p* = 0.001) or 460.3 MET-minutes/week (95% CI: 71.3 to 849.3; *p* = 0.021). Moderate-to-vigorous physical activity (MVPA) increased by a mean of 271.9 MET-minutes/week (95% CI: 6.3 to 537.6; *p* = 0.045) during this period. From baseline to week 12, the mean increase in total PA was 22.7 min/week (95% CI: 4.8 to 40.6; *p* = 0.013) or 465.4 MET-minutes/week (95% CI: 106.7 to 824.1; *p* = 0.011). MVPA increased by a mean of 402.9 MET-minutes/week (95% CI: 129.9 to 676.0; *p* = 0.004). These increases were largely maintained at month 8; at this time point there was a near significant increase in total PA (mean increase 18.6 min/week, 95% CI: − 0.1 to 37.2; *p* = 0.052) and a significant increase in MVPA (mean increase 420.5 MET-minutes/week, 95% CI: 56.4 to 784.6; *p* = 0.024).

The interviews helped to explain why self-reported PA increased but there were no significant changes in steps. Many participants reported making changes to their usual activity type, which would not have been reflected in step count data. For example, some individuals had begun boxing or water-based activities (where it was not practical to wear the Fitbit®), and others reported more gym activity and strength training:

*“One of the complaints that people say is, look, I go in the gym, I work really hard, but it doesn’t record that as a step. I can see that; you’re not really stepping. But it almost looks like you’re not doing any exercise. Some people are doing 5,000 steps a day, but they go in the gym for two hours and that’s not recorded.”*

P6 (Police Community Support Officer, male, age 40+).

The interview findings also explained how the intervention worked, and revealed differences in its impact according to baseline activity levels. The main behaviour change mechanisms that were apparent from the interviews included goal-setting, self-monitoring, awareness, feedback and self-regulation. For example:

*“The goal-setting, I think it made me think about what I wanted to do each week. I would often look at it and go, I need to run one more time this week.”*

P13 (Police sergeant, female, age 18–39).

*“I think the Fitbit raises my awareness to the fact that on certain parts of my shift, I don’t do very much. I can have days where I’m only doing 4,000 steps, 4,500 steps.”*

P4 (Police inspector, male, age 40+).

*“I liked that it all went green when you hit your target. It sounds really silly, but it does make you want to do it because you want to hit it to go green, and it also - when you hit your 10,000 steps, it says, “Congratulations” or “Wow”. It’s just a little message to say well done. I thought that was good.”*

P14 (Police staff, female, age 18–39).

These mechanisms were most pronounced for participants who were less active at baseline (i.e. baseline steps < 10,000/day and IPAQ classification ‘low’ or ‘moderate’). This group perceived the greatest impact of the intervention on their motivation and behaviour. For example:

*“It really has made me think about my lifestyle, my activity or lack of, being conscious of, if I’ve had a really lazy day, I need to do something. I’m not going to get active sat in an armchair or at my desk.”*

P9 (Police staff, female, age 40+, ‘low activity level’* at baseline)

*according to IPAQ classification, equating to less than 600 MET-minutes/week.

In contrast, officers and staff who were highly active and intrinsically motivated at baseline perceived that the technology helped them to maintain, rather than significantly increase, their activity:

*“I’ve always done physical activity. So for me, it’s been a good recording tool but it’s not really made me do any more because I do it anyway and I’ll probably always do it.”*

P6 (Police Community Support Officer, male, age 40+, ‘high activity level’* at baseline) *according to IPAQ classification, equating to approximately 1500–3000 MET-minutes/week.

Longer-term behaviour change was also apparent, particularly for the less active officers and staff. Some participants reported changes in mind set regarding PA, and others showed improved confidence and self-efficacy:

*“It certainly helped in getting me motivated and getting back into being fit again. I’ve got back into that mind set now.”*

P13 (Police sergeant, female, age 18–39).

*“Because of the goals that I’ve achieved since I’ve worn it [the Fitbit], I do feel more confident.”*

P3 (Police sergeant, male, age 18–39).

There was also evidence of habit formation including both wearing the Fitbit® and adopting and maintaining new PA routines:

*“I see no reason why I would not wear it [the Fitbit]. It’s just a bit of a habit now to always check and see how much I’ve slept and I like to know what my heart rate is when I’m out running and so on.”*

P2 (Police constable, male, age 18–39).

While there was a slight reduction in sedentary time during the study, the changes were not statistically significant (see Table 4). The interviews explained that this was mainly due to perceived pressure of work and organisational culture or social norms where breaks were not perceived as appropriate. Officers and staff wished for more opportunities to take breaks during the working day, in addition to encouragement from managers or supervisors:

*“Well, because of the job I’ve got, it’s quite difficult to-, if I said to my supervisor, “My Fitbit tells me I’ve got to get up and do 250 paces”, I don’t know how well that would go down.”*

P9 (Police staff, female, age 40+).

Subgroup analysis revealed no significant differences in changes in PA or sedentary time according to age, gender or occupation (all *p*-values > 0.05).

### Impact on secondary outcomes

Overall, there were no statistically significant changes in physical or mental health-related quality of life, perceived stress, or any of the HPQ outcomes (absenteeism, presenteeism and combined productivity score) from baseline to week 6 (mid-intervention) or baseline to week 12 (post-intervention). From baseline to 8-month follow-up, there was a significant improvement in mental health-related quality of life (mean increase in SF-12 Mental Component Score or MCS 1.75 points, 95% CI: 0.28 to 3.23; *p* = 0.020). Changes in physical health-related quality of life, perceived stress and the HPQ outcomes were non-significant (all p-values > 0.05).

However, the majority of interviewees reported that they perceived wider benefits of using the Fitbit® and Bupa Boost app and/or increasing their PA level, in both the short and longer term. These included weight loss, improved sleep, feeling fitter (including reduced resting heart rate), feeling healthier and having more energy, improved mood, feeling less stressed, and improved resilience.

Some interviewees noticed improved morale and a sense of camaraderie within the organisation, which was reported to result from the social aspects of the intervention (e.g. social support and competitions). For example:

*“I feel there are benefits to having them [Fitbits]... that camaraderie and competitiveness between the team, to outstep each other, do that actual run, or do that extra time of physical activity. I think it’s all useful and increases morale.”*

P2 (Police constable, male, age 18–39).

The analysis of sickness absence data was infeasible due to a lack of complete and accurate data from staff records.

### Engagement and acceptability

Usage data showed that engagement with the Fitbit® was high in both the short and longer term, compared with lower engagement with the Bupa Boost app which declined more rapidly over time. As shown in Table 5, 83% of participants reported wearing the Fitbit® at month 8 compared with only 27% who were still using Bupa Boost at the same time point. The mean wear time for the Fitbit® was 6.6 ± 1.0 days per week for 22.0 ± 3.7 h per day at week 12, and 6.5 ± 1.1 h per week for 21.4 ± 4.1 h per day at month 8. Of the Bupa Boost app users, the majority logged in for one to 5 min per day.

**Table 5:** Self-reported Fitbit® wear and Bupa Boost use

Time point	Number of respondents	Number (%) of participants reporting wearing the Fitbit®	Number (%) of participants reporting using the Bupa Boost app
Week 6	159	156 (98%)	104 (65%)
Week 12	151	146 (97%)	91 (60%)
Month 8	143	119 (83%)	39 (27%)

Note: Participants who reported wearing the Fitbit® or using the Bupa Boost app for any amount of time are included

Usability and usefulness ratings indicated that the Fitbit® was perceived as more user friendly and useful in promoting PA than the Bupa Boost app. Ratings at week 12 are shown in Table 6.

**Table 6:** Perceived usability and usefulness of the Fitbit® and Bupa Boost app at week 12 (post-intervention)

Intervention component	Usability rating			Usefulness rating		
	n	Mean (SD)	Range	n	Mean (SD)	Range
Fitbit® activity monitor	147	4.7 (0.5)	3 to 5	147	3.9 (1.0)	1 to 5
Bupa Boost app	118	3.6 (1.2)	1 to 5	117	3.2 (1.3)	1 to 5

Note: *SD* Standard Deviation; *n* number of responses

Participants were asked: “On a scale of 1 to 5, where 5 = strongly agree, 4 = agree, 3 = neutral, 2 = disagree, 1 = strongly disagree, how much do you agree that the *Fitbit / Bupa Boost* was easy to use [usability] / helped you to be more physically active [usefulness]?”

In accordance with the quantitative results, all of the interviewees stated that the Fitbit® was easy to use and navigate, and that the device had met or exceeded their expectations. In comparison, the Bupa Boost app was seen as more difficult to use and less useful in helping officers and staff to be more active. Participants reported problems linking the app to the Fitbit® and finding colleagues, and perceived that there were too many meaningless notifications and not enough automated tracking of activity within Bupa Boost. Rewards and competitions were also perceived as unfair, for example, points could be earned for goals that were perceived as meaningless:

“The competition of people trying to accumulate points is ridiculous when someone’s ticking a box to say, ‘Be grateful’ or ‘Declutter’. What does that even mean?”

P4 (Police inspector, male, age 40+).

Participants who did not find the Bupa Boost app easy to use or useful tended to use it less frequently or stopped using it altogether. For example:

*“The Bupa Boost app, I used very occasionally because I didn’t particularly find it a very user friendly or useful app.”*

P14 (Police staff, female, age 18–39).

*“I don’t tend to use the Bupa Boost app. I don’t find it very helpful.”*

P3 (Police sergeant, male, age 18–39).

Many participants felt that there was duplication in function between the Fitbit® (and the Fitbit® app) and the Bupa Boost app:

*“It almost became like doing the same thing twice.”*

P12 (Police constable, male, age 40+).

Regarding practicality of the Fitbit®, the device was seen as practical to wear with the police uniform as it was small and lightweight, but also durable. The most frequently suggested improvement was waterproofing:

*“I don’t like the fact that it’s not waterproof. I go to the beach a few days in the summer, surfing, and playing on the beach and in the sea. I’m in the sea for maybe four or five hours. It seems ironic that you’ve got to take off an activity tracker. It seems like it’s almost not fit for purpose.”*

P4 (Police inspector, male, age 40+).

It was also suggested that the algorithm for capturing activity data could be adapted for night shift workers. As the current cut-off for measuring daily steps is midnight to midnight, this is designed for those with a typical 9 to 5 work pattern. This may be an important consideration for tailoring of mHealth technologies:

*“Sometimes … my body clock isn’t midnight to midnight. My body is seven in the morning until seven in the morning. If I do a night shift, I might sleep most of the day, then do a night shift, it will read for that day only 3,000 steps. Of course, I’m going to be awake another 12 hours yet. If you’re a night worker, the data gives you a midnight cut-off, even though you’re going to be awake for another 10 hours.”*

P4 (Police inspector, male, age 40+).

There were large individual differences in levels of engagement with the intervention over time. For many, engagement was consistently high through the 8 months of the study, while others reported fluctuations in their engagement over time. For example, one police officer stopped using the Bupa Boost app but then experienced a motivational pull to use it again:

*“I really missed not going on the app, updating and getting my points up. I find it quite a good motivational tool. So I went back to it about two weeks after stopping.”*

P15 (Police constable, male, age 18–39).

Another officer had stopped wearing the Fitbit® as she felt that it had already helped her to be more active and so was no longer needed, but reported that she would use the device again in the event of a relapse in behaviour:

*“If I slip again, I’d probably put [the Fitbit] on and wear it every day again.”*

P13 (Police sergeant, female, age 18–39).

Although experiences of the intervention were positive overall, the qualitative data highlighted some potential negative consequences of mHealth and fitness technology use for a small number of individuals. Adverse physical effects included skin irritation as a result of Fitbit® wear, which was reported by five participants (approximately 3%). Negative psychological consequences were also reported by a small number of participants, and led two to withdraw from the study. These included feelings of failure and guilt when not meeting goals, and anxiety and cognitive rumination resulting from tracking activity and sleep. For example:

*“The trouble is you look at it and then you get overly anxious about how bad your sleep is. And then that actually can have quite a negative effect because then you’re thinking, ‘Oh, God, I’m not going to get much sleep tonight.’ Or you look at it and go, ‘Oh, I haven’t got much sleep, so therefore, I feel tired.’ I think fitness watches are great, but sometimes it can have quite, I think, a negative impact when you look at your results because you’re overthinking it.”*

P8 (Police constable, female, age 18–39).

### Preferred and impactful intervention components

The study found no differential impact of the ‘individual’ and ‘social’ phases on steps, self-reported PA or sedentary time, in either the short or longer term (see Additional File 5). No significant subgroup differences were observed between the two phases of the intervention by gender, age group, baseline activity level or occupation. According to the post-intervention survey, the majority of participants preferred the ‘individual’ (56%) phase of the study to the ‘social’ (7%) phase. The remaining 37% reported having no preference.

The interviews confirmed that the individual components (including goal-setting, self-monitoring and awareness) were generally perceived as more acceptable and most impactful for increasing motivation and changing behaviour. Some participants reported having concerns regarding privacy and sharing of their PA and health data with their colleagues within the social phase. Others stated that they would prefer to compare and compete with those of a similar age and activity level to themselves:

*“I think a lot of them that do the fitness stuff, they’re lot younger than me and are probably a lot more competitive. It’s probably a bit of an age thing. I couldn’t really be bothered with competing with somebody who’s 25, who’s done 30,000 steps and you know, who thinks it’s really exciting. It just doesn’t do anything for me.”*

P10 (Police constable, female, age 40+).

However, there were large individual differences in preferences and perceived impact of the individual and social components. These appeared to be due to personal preferences and personality differences, rather than associated with any identifiable characteristics such as occupation or baseline activity level:

“I am not into the social aspect of it. It suits certain people… it certainly doesn’t really suit me that much.”

P12 (Police constable, male, age 40+)

“That’s my nature. I’m very competitive. When I’m at work, I get very competitive [laughs]. When it came to the competing against each other… there was that stage where I pushed myself.”

P7 (Police Community Support Officer, female, age 18–39)

## Discussion

The findings of this mixed methods pilot study indicated that an mHealth intervention consisting of a Fitbit® activity monitor and a smartphone app (Bupa Boost) was acceptable and potentially impactful for increasing the PA levels of police officers and staff. There was some evidence that the intervention resulted in behaviour change in both the short and longer term. According to the qualitative findings, this was more pronounced for officers and staff who were less active at the beginning of the study, and lacking in intrinsic motivation. Similar findings have been reported in previous mHealth studies, where less active subgroups have shown the largest increases in PA [49, 50]. This finding is encouraging in that less active individuals are likely to realise the greatest health gains from increasing their PA levels [51].

The quantitative and qualitative findings indicated a lack of an impact of the intervention on sedentary time. The interviews revealed the important influence of contextual factors on sedentary time in the workplace. Perceived pressure of work and organisational culture appeared to be the most prominent barriers to reducing sedentary time; these issues are common amongst desk-based workers [52]. Officers and staff expressed a need for more opportunities to take breaks, and further encouragement from managers or supervisors.

There was a lack of evidence of a difference in impact of the individual and social phases of the study. This is in contrast to previous studies that have reported a larger impact of social app features on PA levels in comparison with other app features [34, 53]. Although the majority of officers and staff preferred the individual intervention components, there were large individual differences in preferences and perceived impact, suggesting the importance of personalisation and tailoring. Tailoring is recognised as a major advantage of mHealth interventions [54] and the need for tailoring of such interventions for specific occupational groups has previously been identified [55].

Engagement with the intervention was moderately high overall, but the Bupa Boost app was associated with lower and more rapidly declining use compared with the Fitbit®. There were clear links between engagement (as defined by use) and perceived usability and usefulness. Levels of engagement throughout the intervention varied greatly between individuals and fluctuated over time. This should not necessarily be seen negatively; ‘effective engagement’ (sufficient engagement to achieve desired outcomes) may be more important than continued high use in digital behaviour change interventions [56]. The present study affirmed this; in a small number of cases, participants perceived that continued use of the Fitbit® and the Bupa Boost app was not necessary to sustain behaviour change.

While the experience of activity tracking was positive for the majority of officers and staff, the study highlighted the potential negative consequences of technology use. This included both physical adverse effects (such as skin irritation due to Fitbit® wear) and psychological consequences (including feelings of failure and guilt when goals were not met, and anxiety and cognitive rumination resulting from activity and sleep tracking). This study is one of only a few to explore these qualitatively. A previous study of activity tracker use in young adults with depression and anxiety reported feelings of guilt and increased anxiety [57]. Another qualitative study found evidence of ‘unhealthy preoccupation’ and ‘obsession’ relating to health and fitness app use in college students [58]. It is important to consider and prepare for such outcomes in future mHealth trials in different populations.

Evidence relating to the impact of digital interventions on wider health and wellbeing outcomes and productivity in a workplace setting is extremely limited [21, 59]. Although the present study found a lack of quantitative evidence for an impact on such outcomes (with the exception of a small improvement in mental health-related quality of life), the interviews were informative in that they elucidated a number of potential benefits that should be explored in future studies. It was not feasible to include sickness absence as an outcome due to issues with data quality and completeness. It is not known whether this was unique to the police forces involved in this study or if this is a more widespread issue, but this will need to be resolved for future studies.

### Strengths and limitations

The study had several strengths, including the use of mixed methods. This produced a more comprehensive and valid picture in relation to not only impact, but also the important aspects of engagement and acceptability, which have been understudied in digital health and mHealth [31, 54]. Additional strengths were the inclusion of multiple outcomes (including both objective and self-reported measures of PA) and the capture of data at multiple time points, which enabled a more detailed exploration of impact and acceptability of the intervention. Furthermore, the study was of relatively long duration with follow-up at 8 months; previous studies of mHealth interventions for PA in other workplace settings have been characterised by short-term interventions and follow-up, generally less than 6 months [22–30]. The high ecological validity of this study, conducted in a real-world setting, and the relatively large, diverse and representative sample of officers and staff (including both urban and rural sites) should also be recognised.

The main limitation of the study was the use of an uncontrolled pre-post design. This was the only feasible design for practical reasons (for example, the Bupa Boost app was being widely promoted across the police forces at the time of the study). The potential confounding of changes in PA levels by temporal and seasonal factors should not be ignored, as there is good evidence that individuals tend to be most active in spring and summer [60]. However, the fact that activity levels increased in the present study despite the baseline measures being taken in summer, the 12-week follow-up in autumn and the 8-month follow-up in winter adds support to suggest a positive impact of the intervention. The potential for a reactivity effect should be considered, i.e. participants might have consciously or subconsciously increased their PA level at baseline in response to wearing the Fitbit®. However, studies have found little evidence for this effect when ‘sealed’ devices with no visual feedback are used (69, 70) as was used in this study. Additional measures taken to improve the rigour of the study included the capture of potential confounders (self-reported data on factors influencing PA levels), the incorporation of qualitative data, and use of multiple outcomes and time points [61, 62].

The decision to use the Fitbit® device itself rather than scientific accelerometers to capture the primary PA outcome may be questioned. Consultations with the Devon and Cornwall Police Health and Wellbeing Board indicated that wearing an accelerometer in addition to the Fitbit® would be considered burdensome, particularly for officers who already have to wear a uniform and carry weighty equipment. Fitbit® data on step count is known to be valid and reliable compared with research-grade accelerometers across different settings [37, 38].

Finally, it is recognised that the setting of the study (police forces in South West England) is specific, therefore the findings may not be generalisable to other police forces in the UK or internationally. Nevertheless, this study provides important and novel contributions to the literature on mHealth and workplace wellness, and the findings have been used to inform the health and wellbeing strategy and practice in the participating police forces. Future studies should focus on a wider range of workplace settings and populations, such as metropolitan police forces or other emergency services.

## Conclusions

This study of an mHealth intervention to promote PA and reduce SB in the police force adds to the limited evidence base on the use of mHealth technology in workplace settings. The findings suggest that a Fitbit® activity monitor and smartphone app are generally acceptable and useful for promoting PA amongst officers and staff, but are less useful in reducing SB. It is recommended that future intervention studies should take a similar mixed methods approach, focus on the long-term impact and acceptability of mHealth in a wider range of workplace settings, and further explore the relative impact of standalone mHealth versus multi-component interventions. Studies should aim to elucidate the wider impact of mHealth interventions on health, wellbeing, productivity and sickness absence. Finally, the use of tailored interventions with different features for specific groups of workers or individuals should be explored. It is anticipated that the findings will provide a valuable guide for designers of activity monitors and apps, those who are involved in the implementation of workplace activity programmes, and future researchers.

## Supplementary Information

**Additional file 1.** Coded behaviour change techniques (BCTs) and intervention components within the Fitbit® activity monitor and Bupa Boost app.

**Additional file 2.** Comparison of PAW-Force study participants from the Plymouth BCU and North Dorset sites with the wider Plymouth BCU and North Dorset police populations: numbers in occupational roles, gender and ethnicity.

**Additional file 3.** Characteristics of interview participants.

**Additional file 4.** Sensitivity analysis for change in mean daily steps, including only participants who reported no events affecting their PA level (e.g. illness, annual leave) in the 7 days prior to data collection.

**Additional file 5.** Comparison of individual and social phase: change in mean daily step count, self-reported physical activity (PA) and sedentary time (all participants).

## Data Availability

The datasets used and/or analysed during the current study are available from the corresponding author on reasonable request.

## Abbreviations

95% CI: 95% Confidence Interval
BCT: Behaviour Change Technique
BCU: Basic Command Unit
BMI: Body Mass Index
CALO-RE taxonomy: Coventry, Aberdeen and London – Refined taxonomy
CVD: Cardiovascular Disease
HPQ: Health and Work Performance Questionnaire
IPAQ: International Physical Activity Questionnaire
mHealth: Mobile Health
MET: Metabolic Equivalent of Task
MVPA: Moderate-to-Vigorous Physical Activity
PA: Physical Activity
PAW-Force: Physical Activity Wearables in the Police Force
PCSO: Police Community Support Officer
PSS-4: Perceived Stress Scale (4-item)
SB: Sedentary Behaviour
SD or ±: Standard Deviation
WHO: World Health Organisation

## References

[1] Ramey SL, Perkhounkova Y, Moon M, Tseng HC, Wilson A, Hein M (2014). Physical activity in police beyond self-report. J Occup Environ Med.

[2] Caneppele S, Aebi MF (2017). Crime drop or police recording flop? On the relationship between the decrease of offline crime and the increase of online and hybrid crimes. Policing.

[3] Ramey SL, Perkhounkova Y, Hein M, Chung S, Franke WD, Anderson AA (2016). Building resilience in an urban police department. J Occup Environ Med.

[4] Ruiz J, Morrow E (2005). Retiring the old centurion: life after a career in policing—an exploratory study. Int J Publ Admin.

[5] Soroka A, Sawicki B (2014). Physical activity levels as a quantifier in police officers and cadets. Int J Occup Med Environ Health.

[6] Can SH, Hendy HM (2014). Behavioral variables associated with obesity in police officers. Ind Health.

[7] Leischik R, Foshag P, Strauß M, Littwitz H, Garg P, Dworrak B (2015). Aerobic capacity, physical activity and metabolic risk factors in firefighters compared with police officers and sedentary clerks. PLoS One.

[8] Anderson AA, Yoo H, Franke WD (2016). Associations of physical activity and obesity with the risk of developing the metabolic syndrome in law enforcement officers. J Occup Environ Med.

[9] Franke WD, Ramey SL, Shelley MC (2002). Relationship between cardiovascular disease morbidity, risk factors, and stress in a law enforcement cohort. J Occup Environ Med.

[10] Hartley TA, Burchfiel CM, Fekedulegn D, Andrew ME, Violanti JM (2011). Health disparities in police officers: comparisons to the U.S. general population. Int J Emerg Ment Health..

[11] Zimmerman FH (2012). Cardiovascular disease and risk factors in law enforcement personnel: a comprehensive review. Cardiol Rev.

[12] Violanti JM, Fekedulegn D, Hartley TA, Andrew ME, Gu JK, Burchfiel CM (2013). Life expectancy in police officers: a comparison with the U.S. general population. Int J Emerg Ment Health.

[13] Guo X, Coberley C, Pope JE, Wells A (2015). The value of a well-being improvement strategy: longitudinal success across subjective and objective measures observed in a firm adopting a consumer-driven health plan. J Occup Environ Med.

[14] Baicker K, Cutler D, Song Z (2010). Workplace wellness programs can generate savings. Health Aff (Millwood).

[15] Lagestad P, Van Den Tillaar R (2014). Longitudinal changes in the physical activity patterns of police officers. Int J Police Sci Manag.

[16] Sullivan AN, Lachman ME (2016). Behavior change with fitness Technology in Sedentary Adults: a review of the evidence for increasing physical activity. Front Public Health.

[17] Teyhen DS, Aldag M, Edinborough E, Ghannadian JD, Haught A, Kinn J (2014). Leveraging technology: creating and sustaining changes for health. Telemed J E Health.

[18] Fanning J, Mullen SP, McAuley E (2012). Increasing physical activity with mobile devices: a meta-analysis. J Med Internet Res.

[19] Bort-Roig J, Gilson ND, Puig-Ribera A, Contreras RS, Trost SG (2014). Measuring and influencing physical activity with smartphone technology: a systematic review. Sports med (Auckland, NZ).

[20] Afshin A, Babalola D, McLean M, Yu Z, Ma W, Chen CY (2016). Information technology and lifestyle: a systematic evaluation of internet and Mobile interventions for improving diet, physical activity, obesity, tobacco, and alcohol use. J Am Heart Assoc.

[21] Buckingham SA, Williams AJ, Morrissey K, Price L, Harrison J (2019). Mobile health interventions to promote physical activity and reduce sedentary behaviour in the workplace: a systematic review. Digit Health..

[22] Ganesan AN, Louise J, Horsfall M, Bilsborough SA, Hendriks J, McGavigan AD (2016). International Mobile-health intervention on physical activity, sitting, and weight: the Stepathlon cardiovascular health study. J Am Coll Cardiol.

[23] Neil-Sztramko SE, Gotay CC, Sabiston CM, Demers PA, Campbell KC (2017). Feasibility of a telephone and web-based physical activity intervention for women shift workers. Transl Behav Med.

[24] Patel MS, Volpp KG, Rosin R, Bellamy SL, Small DS, Heuer J (2018). A randomized, controlled trial of lottery-based financial incentives to increase physical activity among overweight and obese adults. Am J Health Promot.

[25] Simons D, De Bourdeaudhuij I, Clarys P, De Cocker K, Vandelanotte C, Deforche B (2018). Effect and process evaluation of a smartphone app to promote an active lifestyle in lower educated working young adults: cluster randomized controlled trial. JMIR mHealth uHealth..

[26] Olsen HM, Brown WJ, Kolbe-Alexander T, Burton NW (2018). A brief self-directed intervention to reduce office Employees' sedentary behavior in a flexible workplace. J Occup Environ Med.

[27] Poirier J, Bennett WL, Jerome GJ, Shah NG, Lazo M, Yeh HC (2016). Effectiveness of an Activity Tracker- and Internet-Based Adaptive Walking Program for Adults: A Randomized Controlled Trial. J Med Internet Res.

[28] Reed JL, Cole CA, Ziss MC, Tulloch HE, Brunet J, Sherrard H (2018). The impact of web-based feedback on physical activity and cardiovascular health of nurses working in a cardiovascular setting: a randomized trial. Front Physiol.

[29] Skogstad M, Lunde LK, Skare O, Mamen A, Alfonso JH, Ovstebo R (2016). Physical activity initiated by employer and its health effects; an eight week follow-up study. BMC Public Health.

[30] van Dantzig S, Geleijnse G, van Halteren AT (2013). Toward a persuasive mobile application to reduce sedentary behavior. Pers Ubiquitous Comput.

[31] McCallum C, Rooksby J, Gray CM (2018). Evaluating the impact of physical activity apps and Wearables: interdisciplinary review. JMIR mHealth uHealth..

[32] Buijink AW, Visser BJ, Marshall L (2013). Medical apps for smartphones: lack of evidence undermines quality and safety. Evid Based Med.

[33] Knight E, Stuckey MI, Prapavessis H, Petrella RJ (2015). Public health guidelines for physical activity: is there an app for that? A review of android and apple app stores. JMIR mHealth uHealth..

[34] King AC, Hekler EB, Grieco LA, Winter SJ, Sheats JL, Buman MP (2016). Effects of three motivationally targeted Mobile device applications on initial physical activity and sedentary behavior change in midlife and older adults: a randomized trial. PLoS One.

[35] Harries T, Eslambolchilar P, Rettie R, Stride C, Walton S, van Woerden HC (2016). Effectiveness of a smartphone app in increasing physical activity amongst male adults: a randomised controlled trial. BMC Public Health.

[36] Michie S, Ashford S, Sniehotta FF, Dombrowski SU, Bishop A, French DP (2011). A refined taxonomy of behaviour change techniques to help people change their physical activity and healthy eating behaviours: the CALO-RE taxonomy. Psychol Health.

[37] Evenson KR, Goto MM, Furberg RD (2015). Systematic review of the validity and reliability of consumer-wearable activity trackers. Int J Behav Nutr Phys Act.

[38] Feehan LM, Geldman J, Sayre EC, Park C, Ezzat AM, Yoo JY (2018). Accuracy of Fitbit devices: systematic review and narrative syntheses of quantitative data. JMIR mHealth uHealth..

[39] Tedesco S, Sica M, Ancillao A, Timmons S, Barton J, O'Flynn B (2019). Validity evaluation of the Fitbit Charge2 and the Garmin vivosmart HR+ in free-living environments in an older adult cohort. JMIR mHealth uHealth..

[40] Wang JB, Cataldo JK, Ayala GX, Natarajan L, Cadmus-Bertram LA, White MM (2016). Mobile and wearable device features that matter in promoting physical activity. J Mob Technol Med.

[41] Craig CL, Marshall AL, Sjostrom M, Bauman AE, Booth ML, Ainsworth BE (2003). International physical activity questionnaire: 12-country reliability and validity. Med Sci Sports Exerc.

[42] Ware J, Kosinski M, Keller SD (1996). A 12-item short-form health survey: construction of scales and preliminary tests of reliability and validity. Med Care.

[43] Cohen S, Kamarck T, Mermelstein R (1983). A global measure of perceived stress. J Health Soc Behav.

[44] Kessler RC, Barber C, Beck A, Berglund P, Cleary PD, McKenas D (2003). The World Health Organization health and work performance questionnaire (HPQ). J Occup Environ Med.

[45] Patton MQ (2001). Qualitative research & evaluation methods.

[46] StataCorp (2017). Stata Statistical Software: Release 15.

[47] Ritchie J, Spencer L, Bryman A, Burgess RG (1994). Qualitative data analysis for applied policy research. Analysing qualitative data.

[48] QSR International. NVivo qualitative data analysis software: version 11: QSR International Pty Ltd.; 2015.

[49] Schrager JD, Shayne P, Wolf S, Das S, Patzer RE, White M (2017). Assessing the influence of a Fitbit physical activity monitor on the exercise practices of emergency medicine residents: a pilot study. JMIR mHealth uHealth.

[50] Xian Y, Xu H, Xu H, Liang L, Hernandez AF, Wang TY (2017). An Initial Evaluation of the Impact of Pokemon GO on Physical Activity. J Am Heart Assoc.

[51] Blair SN, Connelly JC (1996). How much physical activity should we do? The case for moderate amounts and intensities of physical activity. Res Q Exercise Sport.

[52] Cole JA, Tully MA, Cupples ME (2015). "they should stay at their desk until the work's done": a qualitative study examining perceptions of sedentary behaviour in a desk-based occupational setting. BMC Res Notes.

[53] Hamari J, Koivisto J (2015). "working out for likes": an empirical study on social influence in exercise gamification. Comput Hum Behav.

[54] Huang Y, Benford S, Blake H (2019). Digital interventions to reduce sedentary behaviors of office workers: scoping review. J Med Internet Res.

[55] Gilson ND, Pavey TG, Wright OR, Vandelanotte C, Duncan MJ, Gomersall S (2017). The impact of an m-health financial incentives program on the physical activity and diet of Australian truck drivers. BMC Public Health.

[56] Yardley L, Spring BJ, Riper H, Morrison LG, Crane DH, Curtis K (2016). Understanding and promoting effective engagement with digital behavior change interventions. Am J Prev Med.

[57] Kanstrup AM, Bertelsen P, Jensen MB (2018). Contradictions in digital health engagement: an activity tracker's ambiguous influence on vulnerable young adults' engagement in own health. Digit Health..

[58] Gowin M, Cheney M, Gwin S, Wann TF (2015). Health and fitness app use in college students: a qualitative study. Am J Health Educ.

[59] Howarth A, Quesada J, Silva J, Judycki S, Mills PR (2018). The impact of digital health interventions on health-related outcomes in the workplace: a systematic review. Digit Health.

[60] O'Connell SE, Griffiths PL, Clemes SA (2014). Seasonal variation in physical activity, sedentary behaviour and sleep in a sample of UK adults. Ann Hum Biol.

[61] Craig P, Cooper C, Gunnell D, Haw S, Lawson K, Macintyre S (2012). Using natural experiments to evaluate population health interventions: new Medical Research Council guidance. J Epidemiol Community Health.

[62] Moore G, Audrey S, Barker M, Bond L, Bonell C, Hardeman W (2014). Process evaluation of complex interventions: UK Medical Research Council (MRC) guidance.

