# Supplementary material for: The Physical Activity Wearables in the Police Force (PAW-Force) study: acceptability and impact
Source: BMC Public Health. 2020 Nov 3;20:1645. doi: 10.1186/s12889-020-09776-1 (PMC7607613; doi:10.1186/s12889-020-09776-1)
Supplement: Supplementary file 1 — Additional file 1. Coded behaviour change techniques (BCTs) and intervention components within the Fitbit® activity monitor and Bupa Boost app. [file 12889_2020_9776_MOESM1_ESM.docx]

**Additional File 1**

**Coded behaviour change techniques (BCTs) and intervention components within the Fitbit^®^ activity monitor and Bupa Boost app**

| **Specific behaviour change technique (BCT, CALO-RE taxonomy)** | **Included in Fitbit^®^?**  **(S = Social phase only)** | **Component or feature** | **Included in Bupa Boost app?**  **(S = Social phase only)** | **Component or feature** |
| --- | --- | --- | --- | --- |
| 1. Provide information on consequences of behaviour in **general** | N |  | Y | Self-help information on PA and health (Bupa library) |
| 1. Provide information on consequences of behaviour to the **individual** | N |  | N |  |
| 1. Provide information about others’ approval | Y***** (S) | Messaging with friends (approval or disapproval). | Y (S) | Messaging with colleagues (approval or disapproval).  ‘Likes’ for others’ achievements. |
| 1. Provide normative information about others’ behaviour | Y***** (S) | Compare weekly steps and achievements with friends within app. | Y (S) | Social feed – compare daily PA and goals achieved with colleagues.  Leader board – compare wellness points and achievements with colleagues (weekly, monthly, all time). |
| 1. Goal setting (behaviour) | Y | Set activity goals in app, e.g. 10,000 daily steps, 30 daily active minutes, 250 steps per hour. | Y | Set general or specific PA/fitness goals (choose from suggested list or custom, e.g. walk to work). Also nutrition, mindfulness and relaxation (if desired). |
| 1. Goal setting (outcome) | Y | Set weight goals in app (if desired) | N |  |
| 1. Action planning | Y | Activity goals specified in terms of context, frequency, duration or intensity – e.g. 10,000 daily steps. | Y | Set specific activity goals in app – e.g. walk to work on three days per week. |
| 1. Barrier identification / problem solving | N |  | N |  |
| 1. Set graded tasks | Y | Small goals of 250 steps per hour – helps to meet overall daily step goal. | N |  |
| 1. Prompt review of behavioural goals | Y | Detail of goals achieved given in app. Goals can be edited at any time. | Y | Tick off goals in app when achieved. Goals achieved are listed in ‘activity feed’. Goals can be edited at any time. |
| 1. Prompt review of outcome goals | Y | Weight goals may be reviewed and edited at any time (if desired) | N |  |
| 1. Provide rewards contingent on effort or progress towards behaviour | Y | Virtual badges for PA progress and ‘lifetime achievements’ (e.g. the ‘London Underground’ badge for walking 402 kilometres in total) | Y | Earn wellness points for progress towards behaviour (e.g. earn more points for higher daily steps). Virtual badges for PA progress and ‘lifetime achievements’. |
| 1. Provide rewards contingent on successful behaviour | Y | Virtual badges and trophies for achieving goals. | Y | Wellness points and virtual badges for goals achieved (and goal streaks). |
| 1. Shaping | Y | Graded (virtual) rewards for greater achievements over time. Goal streaks within challenges* | Y | Graded (virtual) rewards for greater achievements over time. Goal streaks. |
| 1. Prompting generalisation of a target behaviour | N |  | N |  |
| 1. Prompt self-monitoring of behaviour | Y | Monitoring of steps, distance, calories burned, floors climbed, active minutes (also biofeedback – sleep and heart rate) | Y | Monitoring of steps and activity duration (via data from Fitbit^®^) |
| 1. Prompt self-monitoring of behavioural outcome | Y | Monitoring of resting heart rate and weight (if desired) | N |  |
| 1. Prompting focus on past success**^1^** | N |  | N |  |
| 1. Provide feedback on performance | Y | Feedback on progress towards goals and goals achieved (in-app and notifications). Includes visual feedback – colour changes to green when activity goals are met. Feedback is real-time and personalised. Can also review past reports of PA and sedentary time in app (also heart rate and sleep if desired) | Y | Feedback on progress towards goals and goals achieved (in-app and notifications). Real-time and personalised. View past reports of PA, goals achieved, and previous wellness points and badges. |
| 1. Provide information on **where and when** to perform the behaviour | N |  | N |  |
| 1. Provide instruction on how to perform the behaviour | N |  | N |  |
| 1. Model / demonstrate the behaviour | N |  | N |  |
| 1. Teach to use prompts / cues | Y | Smart notifications for PA and SB, e.g. reminders to move when 250 steps per hour not reached | Y | Prompts/notifications to remind the individual when a goal has not been achieved. |
| 1. Environmental restructuring | N |  | N |  |
| 1. Agree behavioural contract | N |  | N |  |
| 1. Prompt practice | N |  | N |  |
| 1. Use of follow-up prompts | N | (Prompts are consistent in frequency throughout the intervention and maintenance phase) | N | (Prompts are consistent in frequency throughout the intervention and maintenance phase) |
| 1. Facilitate social comparison | Y***** (S) | Compare weekly steps and achievements with friends within app. Social challenges. | Y (S) | Social feed.  Leader board.  Individual and group competitions / challenges. |
| 1. Plan social support / social change | Y***** (S) | Supportive messaging with friends. | Y (S) | Supportive messaging with colleagues. ‘Likes’ for others’ achievements. |
| 1. Prompt identification as role model / position advocate | N |  | N |  |
| 1. Prompt anticipated regret | N |  | N |  |
| 1. Fear arousal | N |  | N |  |
| 1. Prompt self-talk | N |  | N |  |
| 1. Prompt use of imagery | N |  | N |  |
| 1. Relapse prevention / coping planning | N |  | N |  |
| 1. Stress management / emotional control training | Y | Relaxation exercises within the Fitbit^®^ (guided breathing) | N |  |
| 1. Motivational interviewing | N |  | N |  |
| 1. Time management | N |  | N |  |
| 1. General communication skills training | N |  | N |  |
| 1. Stimulate anticipation of future rewards | Y | User is aware from the outset that (virtual) rewards will be given for progress and achievements. | Y | View available badges and achievements / activities needed to earn them. |

***** = minimal use in this intervention (participants were encouraged to focus on the Bupa Boost app for these behaviour change techniques) **^1^** Review of past reports of PA and sedentary time is not classed as focus on past success as this BCT relates to behaviour preceding the intervention according to the CALO-RE taxonomy

The PAW-Force intervention included 20 of a possible 40 unique BCTs according to the CALO-RE taxonomy. Five BCTs were included in the Fitbit^®^ (or Fitbit^®^ app) only; these were: **goal-setting for outcome; set graded tasks; prompt review of outcome goals; prompt self-monitoring of outcome; stress management training**. One BCT (**provision of information on consequences of behaviour in general**) was included in the Bupa Boost app only. A further 14 BCTs were included in both the Fitbit^®^ and Bupa Boost app:

- **Provide information about others’ approval (social phase only)**
- **Provide normative information about others’ behaviour (social phase only)**
- **Goal-setting (behaviour)**
- **Action planning**
- **Prompt review of behavioural goals**
- **Provide rewards contingent on effort or progress towards behaviour**
- **Provide rewards contingent on successful behaviour**
- **Shaping**
- **Prompt self-monitoring of behaviour**
- **Provide feedback on performance**
- **Teach to use prompts / cues**
- **Facilitate social comparison (social phase only)**
- **Plan social support / social change (social phase only)**
- **Stimulate anticipation of future rewards**
