# Supplementary material for: The Physical Activity Wearables in the Police Force (PAW-Force) study: acceptability and impact
Source: BMC Public Health. 2020 Nov 3;20:1645. doi: 10.1186/s12889-020-09776-1 (PMC7607613; doi:10.1186/s12889-020-09776-1)
Supplement: Supplementary file 2 — Additional file 2. Comparison of PAW-Force study participants from the Plymouth BCU and North Dorset sites with the wider Plymouth BCU and North Dorset police populations: numbers in occupational roles, gender and ethnicity. [file 12889_2020_9776_MOESM2_ESM.docx]

**Additional File 2**

***Comparison of PAW-Force study participants from the Plymouth BCU and North Dorset sites with the wider Plymouth BCU and North Dorset police populations: numbers in occupational roles, gender and ethnicity***

|  | **Plymouth BCU** | | **North Dorset** | |
| --- | --- | --- | --- | --- |
|  | **n (%) in study sample** | **n (%) in Plymouth BCU** | **n (%) in study sample** | **n (%) in North Dorset** |
| Occupation  Police officers | 81 (63) | 654 (52) | 33 (63) | 45 (62) |
| Police staff | 33 (26) | 488 (38) | 3 (6) | 7 (10) |
| PCSOs and special constables  Gender  Male  Female  Ethnicity  White  Other ethnicity  Undisclosed | 14 (11)  73 (57)  55 (43)  125 (98)  2 (2)  1 (1) | 126 (10)  691 (54)  577 (46)  976 (77)  14 (1)  278 (22) | 16 (31)  34 (65)  18 (35)  52 (100)  0 (0)  0 (0) | 21 (29)  48 (66)  25 (34)  70 (96)  2 (3)  1 (1) |
| *Total* | *128 (100)* | *1268 (100)* | *52 (100)* | *73 (100)* |

**Note:** Data provided by Performance & Analysis teams, Devon & Cornwall Police (2017) and Dorset Police (2017)

Percentages may not total 100 due to rounding.
