# Supplementary material for: The Physical Activity Wearables in the Police Force (PAW-Force) study: acceptability and impact
Source: BMC Public Health. 2020 Nov 3;20:1645. doi: 10.1186/s12889-020-09776-1 (PMC7607613; doi:10.1186/s12889-020-09776-1)
Supplement: Supplementary file 3 — Additional file 3. Characteristics of interview participants. [file 12889_2020_9776_MOESM3_ESM.docx]

**Additional File 3**

***Characteristics of interview participants***

| **Interviewee ID** | **Gender** | **Age category** | **Occupation (at baseline)** | **Interviews completed**  **(1 = pre-intervention (week 0),**  **2 = post-intervention (week 12),**  **3 = follow-up (month 8))** |
| --- | --- | --- | --- | --- |
| 1 | M | 18-39 | Police staff | 1 |
| 2 | M | 18-39 | Police officer (constable) | 1,2,3 |
| 3 | M | 18-39 | Police officer (sergeant) | 1,2,3 |
| 4 | M | 40+ | Police officer (inspector) | 1,2,3 |
| 5 | M | 40+ | Police staff | 1 |
| 6 | M | 40+ | PCSO | 1,2,3 |
| 7 | F | 18-39 | PCSO | 1,2 |
| 8 | F | 18-39 | Police officer (constable) | 1,2,3 |
| 9 | F | 40+ | Police staff | 1,3 |
| 10 | F | 40+ | Police officer (constable) | 1,2,3 |
| 11 | M | 18-39 | Police staff and special constable | 2 |
| 12 | M | 40+ | Police officer (constable) | 2 |
| 13 | F | 18-39 | Police officer (sergeant) | 2,3 |
| 14 | F | 18-39 | Police staff | 2,3 |
| 15 | M | 18-39 | Police officer (constable) | 3 |
| 16 | M | 40+ | Police officer (inspector) | 3 |
