# Supplementary material for: The Physical Activity Wearables in the Police Force (PAW-Force) study: acceptability and impact
Source: BMC Public Health. 2020 Nov 3;20:1645. doi: 10.1186/s12889-020-09776-1 (PMC7607613; doi:10.1186/s12889-020-09776-1)
Supplement: Supplementary file 4 — Additional file 4. Sensitivity analysis for change in mean daily steps, including only participants who reported no events affecting their PA level (e.g. illness, annual leave) in the 7 days prior to data collection. [file 12889_2020_9776_MOESM4_ESM.docx]

**Additional File 4**

**Sensitivity analysis for change in mean daily steps, including only participants who reported no events affecting their PA level (e.g. illness, annual leave) in the seven days prior to data collection**

| **Mean change from baseline (week 0)** | | | | | | | | |
| --- | --- | --- | --- | --- | --- | --- | --- | --- |
| **Mid-intervention**  **(end of individual phase, week 6)** | | | **Post-intervention**  **(end of social phase, week 12)** | | | **Follow-up**  **(month 8)** | | |
| **Mean change (SD)**  **n = number of observations** | **95% CI** | **p-value** | **Mean change (SD)**  **n = number of observations** | **95% CI** | **p-value** | **Mean change (SD)**  **n = number of observations** | **95% CI** | **p-value** |
| -272 (2,520)  n = 46 | -1,021 to +476 | 0.467 | +262 (2,561)  n = 57 | -418 to +941 | 0.444 | -765 (3,178)  n = 42 | -1,755 to +225 | 0.126 |

**Note:** SD = Standard Deviation; 95% CI = 95% Confidence Interval. Pairwise deletion used.

p-values where significant (i.e. <0.05) are highlighted in bold
