# Supplementary material for: The Physical Activity Wearables in the Police Force (PAW-Force) study: acceptability and impact
Source: BMC Public Health. 2020 Nov 3;20:1645. doi: 10.1186/s12889-020-09776-1 (PMC7607613; doi:10.1186/s12889-020-09776-1)
Supplement: Supplementary file 5 — Additional file 5. Comparison of individual and social phase: change in mean daily step count, self-reported physical activity (PA) and sedentary time (all participants). [file 12889_2020_9776_MOESM5_ESM.docx]

**Additional File 5**

**Comparison of individual and social phase: change in mean daily step count, self-reported physical activity (PA) and sedentary time (all participants)**

| **Outcome** | **Number of observations^1^** | **Mean change individual phase (baseline to week 6)**  **(SD)** | **Mean change social phase (baseline to week 12)**  **(SD)** | **Mean difference individual vs. social phase^2^ (SD)** | **95% CI for difference** | **p-value for diff.** |
| --- | --- | --- | --- | --- | --- | --- |
| Mean daily step count | 95 | -284 (2,685) | -68 (2,679) | 216 (2,666) | -328 to 759 | 0.433 |
| Total PA (minutes/ week) | 143 | 23.6 (108.6) | 20.9 (113.3) | -2.7 (118.7) | -22.3 to 16.9 | 0.786 |
| Total PA (MET-minutes/ week) | 143 | 363.7 (2,493.6) | 434.0 (2,274.3) | 70.3 (2,507.7) | -344.3 to 484.8 | 0.738 |
| MVPA  (MET-minutes/ week) | 143 | 231.7 (1,720.8) | 371.0 (1,722.3) | 139.4 (1,790.8) | -156.7 to 435.4 | 0.354 |
| Sedentary time (hours on a typical weekday) | 143 | -0.06 (3.38) | 0.08 (3.47) | 0.14 (3.23) | -0.39 to 0.67 | 0.604 |

**Note:** SD = Standard Deviation; 95% CI = 95% Confidence Interval

p-values where significant (i.e. <0.05) are highlighted in bold.

**^1^** Complete case analysis (i.e. only participants with outcome data at baseline, 6 weeks and 12 weeks included)

**^2^** Positive values indicate higher relative increase (or lower decrease) in steps during social phase
